# RNA-Sequencing Analysis Reveals a Regulatory Role for Transcription Factor *Fezf2* in the Mature Motor Cortex

**DOI:** 10.3389/fnmol.2017.00283

**Published:** 2017-09-07

**Authors:** Alison J. Clare, Hollie E. Wicky, Ruth M. Empson, Stephanie M. Hughes

**Affiliations:** ^1^Department of Biochemistry, School of Biomedical Sciences, University of Otago Dunedin, New Zealand; ^2^Brain Health Research Centre, University of Otago Dunedin, New Zealand; ^3^Genetics Otago, University of Otago Dunedin, New Zealand; ^4^Department of Physiology, School of Biomedical Sciences, University of Otago Dunedin, New Zealand

**Keywords:** *Fezf2*, RNA-seq, motor cortex, mouse, lentivirus, shRNA

## Abstract

*Forebrain embryonic zinc finger* (*Fezf2*) encodes a transcription factor essential for the specification of layer 5 projection neurons (PNs) in the developing cerebral cortex. As with many developmental transcription factors, *Fezf2* continues to be expressed into adulthood, suggesting it remains crucial to the maintenance of neuronal phenotypes. Despite the continued expression, a function has yet to be explored for *Fezf2* in the PNs of the developed cortex. Here, we investigated the role of *Fezf2* in mature neurons, using lentiviral-mediated delivery of a shRNA to conditionally knockdown the expression of *Fezf2* in the mouse primary motor cortex (M1). RNA-sequencing analysis of *Fezf2*-reduced M1 revealed significant changes to the transcriptome, identifying a regulatory role for *Fezf2* in the mature M1. Kyoto Encyclopedia Genes and Genomes (KEGG) pathway analyses of *Fezf2*-regulated genes indicated a role in neuronal signaling and plasticity, with significant enrichment of neuroactive ligand-receptor interaction, cell adhesion molecules and calcium signaling pathways. Gene Ontology analysis supported a functional role for *Fezf2*-regulated genes in neuronal transmission and additionally indicated an importance in the regulation of behavior. Using the mammalian phenotype ontology database, we identified a significant overrepresentation of *Fezf2*-regulated genes associated with specific behavior phenotypes, including associative learning, social interaction, locomotor activation and hyperactivity. These roles were distinct from that of *Fezf2*-regulated genes identified in development, indicating a dynamic transition in *Fezf2* function. Together our findings demonstrate a regulatory role for *Fezf2* in the mature brain, with *Fezf2*-regulated genes having functional roles in sustaining normal neuronal and behavioral phenotypes. These results support the hypothesis that developmental transcription factors are important for maintaining neuron transcriptomes and that disruption of their expression could contribute to the progression of disease phenotypes.

## Introduction

The pyramidal projection neurons (PNs) of the mature cortex display vast diversity in their phenotypes and connections (Molyneaux et al., 2007; Spruston, 2008). These excitatory PNs encode messages that are critical for mediating higher cognitive function and behavioral output (Spruston, 2008). Often neurodegenerative disease preferentially affects specific neuronal subtypes, regardless of a universal role for the causative factors (Yang et al., 2014), highlighting that vulnerability can be unique to specific PN types. Therefore, broadening our knowledge of the regulatory factors that maintain neuronal function and diversity is critical to our understanding of the brain in health and disease.

Spatially restricted expression of transcription factors play an important role in directing the fate of distinct PN types in the cortex (Arlotta et al., 2005; Chen J.-G. et al., 2005; Chen B. et al., 2005; Molyneaux et al., 2005; Alcamo et al., 2008; Britanova et al., 2008; McKenna et al., 2011; Kwan et al., 2012; Lodato et al., 2014) and many have continued expression into adulthood (Inoue et al., 2004; Ozdinler et al., 2011; Deneris and Hobert, 2014; Zeisel et al., 2015). This raises the intriguing possibility that these factors play a role in maintaining adult neuronal identity. Certainly, recent studies in mammalian models have demonstrated roles for developmentally important transcription factors in maintaining several distinct neuronal subtypes (Kadkhodaei et al., 2009; Liu et al., 2010; Tsarovina et al., 2010; Song et al., 2011; Domanskyi et al., 2014). For example, the conditional knockdown of *Nurr1*, a key transcription factor for differentiation of midbrain dopaminergic neurons, resulted in a loss of midbrain dopaminergic neurons and striatal dopaminergic neuronal markers in the adult brain, accompanied by impaired motor behavior (Kadkhodaei et al., 2009). The developmental transcription factor, *Pet-1*, is required for 5-HT synthesis in the adult brain and conditional knockout of *Pet-1* elevates anxiety behaviors in mice (Liu et al., 2010). Results such as these highlight the clinical implications of understanding maintenance of neuronal identity. However, despite the importance the cortex has for mediating higher-cognitive behavior, there has been no investigation of the potential regulatory factors maintaining neuronal diversity within the cortex.

During development, highly organized expression of transcription factors are essential to ensure the correct specification of PN types in the layered structure of the cortex (Arlotta et al., 2005; Han et al., 2011; Kwan et al., 2012; Srinivasan et al., 2012). One such transcription factor, *Forebrain embryonic zinc finger 2* (*Fezf2* also known as *Zfp312, Fezl, Znf312*), is expressed in the deep layer PNs (layer 5 and 6), where it is critical to the specification of corticospinal projection neurons (CSpPNs; Chen J.-G. et al., 2005; Molyneaux et al., 2005). The restricted expression of *Fezf2* persists into adulthood (Inoue et al., 2004; Ozdinler et al., 2011; Tantirigama et al., 2016), where high levels of expression in layer 5 were thought to be a CSpPN specific marker (Ozdinler et al., 2011). However, recent work found that *Fezf2*-expression identifies a diverse group of PNs in layer 5 of the mature primary motor cortex (M1; Tantirigama et al., 2016). These comprised of intratelencephalic projection neurons (IT-PNs), which are affected in disorders such as schizophrenia (Shepherd, 2013) and pyramidal tract projection neurons (PT-PNs), including CSpPNs that are lost in the progression of amyotrophic lateral sclerosis (ALS; Ozdinler et al., 2011). The diversity of *Fezf2* expression within layer 5 of M1 makes it an interesting target to investigate for the maintenance of layer-specific PNs in the adult cortex, with the potential to identify target factors with broad clinical implications.

Here, we explored a role for *Fezf2* in maintaining the molecular profiles of mature neurons in the mouse M1. The effects of lentiviral-mediated conditional knockdown of *Fezf2* in the mature M1 were analyzed by RNA-sequencing. We found significant changes in gene expression demonstrating a functional role for *Fezf2* in the mature brain. Through comparison to *Fezf2*-regulated genes previously identified in development (Lodato et al., 2014), we discovered a distinct transition in *Fezf2* function. Bioinformatic analyses of the differentially expressed genes in mature M1 revealed a functional role for *Fezf2* in the regulation of ion flux and cell signaling. In particular, we found *Fezf2* regulates an enrichment of genes important to the functional phenotypes of *Fezf2*-expressing neurons, including genes that regulate calcium transport at the cell membrane. Gene expression changes additionally implicate *Fezf2* in regulating molecular expression profiles that are known to be important for the regulation of behavior. Together, these findings demonstrate an importance for *Fezf2* in maintaining functionally important molecular expression profiles in the mature brain, distinct from its role in development.

## Materials and Methods

### Mice

All experiments were performed with male Swiss-Webster mice of either wild-type (non-transgenic) or hemizygous transgenic (Zfp312-EGFP)CO61GsatMmnc mouse line (Gong et al., 2003) bred on a Swiss-Webster background strain. The transgenic mice express green fluorescence protein (GFP) under the control of *Fezf2* regulatory elements and are referred to from here on as *Fezf2-Gfp* mice. All animal husbandry, surgical procedures and use of animal tissue was approved by the University of Otago Animal Ethics Committee and the use of lentivirus (LV) in the mouse brain (*in vivo*) was approved by the Environmental Protection Authority (EPA), New Zealand under approval number GMD002851.

### Lentiviral Vector Packaging

Lentiviral vectors were generated with approval from the EPA (GMD002849). The mouse *Fezf2 shRNA* and *non-silencing shRNA* were purchased from Open Biosystems (GE Healthcare, Dharmacon, CO, USA) and cloned into a HIV-1 derived lentiviral expression construct (Desmaris et al., 2001) downstream of a rat *Synapsin I* 1.1 kb promoter fragment (Dittgen et al., 2004) and mCherry reporter gene (*pLV.SynI.mCherry.Fezf2shRNA* or *non-silencing shRNA*). The *shRNA* sequences are flanked by endogenous mir-30 sequences, allowing both mCherry and *shRNA* to be transcribed by a Pol-II promoter (Fellmann et al., 2013), in this case *SynI*.

The lentiviral constructs were packaged using a second generation packaging system (Zufferey et al., 1997). Briefly, HEK293FT cells were plated at 5.4 × 10^6^ cells per T75 flask and transfected with 18.6 μg shRNA-expressing construct, 9.6 μg *psPAX2* (gifted from Didier Trono; Addgene plasmid # 12260) and 4.8 μg *pVSVG* (ViraPower™ Lentiviral Packaging Mix, Invitrogen, New Zealand) in Opti-MEM (Life Technologies, New Zealand) containing Lipofectamine-2000 (Life Technologies, New Zealand). The virus-containing media was recovered 48 h post-transfection. Viral particles were concentrated by ultra-centrifugation in a Beckman L-70 (SW28 rotor; Beckman Coulter, Carlsbad, CA, USA) at 110,000 × *g* for 90 min at 4°C and the viral pellet was resuspended in lactose (40 mg/mL in Dulbecco’s phosphate buffered saline) and stored at −80°C (Schoderboeck et al., 2015). The viral genomic titres were determined using qPCR with primers for an enhancer incorporated into the lentiviral vectors; woodchuck posttranscriptional response element (Supplementary Table S1), as previously described in Best et al. (2017). The titres ranged from 7.02 × 10^9^ − 3.72 × 10^10^ genomes/mL.

### Stereotaxic Surgeries for the Injection of Lentivirus

Male mice (P21–25) were anesthetized by a sub-cutaneous injection of 0.05 mg/kg atropine (Baxter Healthcare Ltd.), 0.5 mg/kg domitor (Novartis New Zealand Ltd.) and 70 mg/kg ketamine (Parnell Laboratories New Zealand Ltd.) and secured onto stereotaxic equipment using 45° non-rupture ear bars and a inserting nose clamp gently into the mouth (310037R, Kopf Instruments). A 10 μL Nanofil syringe and 33 G needle assembly (World Precision Instruments, Sarasota, FL, USA) was used to deliver 1 μL of LV through a craniotomy in the skull at two injection sites in the M1; 0.85 mm anterior from Bregma, 1.65 mm lateral from midline and 0.85 mm depth from the pial layer and 0.4 mm anterior from Bregma, 1.4 mm lateral from the midline and 0.6 mm depth from the pial layer.

### Histology

Four weeks after lentiviral injections, mice (P49–53) were anesthetized with pentobarbitol (150 mg/kg) and perfused intracardially with 20 mL ice cold 0.9% (w/v) NaCl followed by 20 mL ice cold 4% (w/v) paraformaldehyde in 0.1 M phosphate buffer (pH 7.2). The brain was post-fixed overnight in 4% (w/v) PFA at 4°C, before cryoprotecting in 30% (w/v) sucrose for 2–3 days at 4°C. Tissue was frozen in optimal cutting temperature medium (OCT), Tissue-Tek^®^ (Thermo Fisher Scientific, New Zealand) before cutting. Coronal sections 40 μm thick were cut at −20°C using a Leica CM3050 cryostat (Leica Biosystems, Lincolnshire, IL, USA). The sections were processed for immunohistochemistry (see Supplementary Methods) to enhance the GFP signal from *Fezf2-Gfp* mouse tissue and the LV reporter gene, mCherry.

### Analysis of Transduction Efficiency *In Vivo*

For transduction efficiency analysis of *Fezf2*-GFP positive cells *in vivo*, three *Fezf2-Gfp* male mice were injected with *pLenti.SynI.mCherry.non-silencingshRNA* and analyzed 4 weeks later. GFP and mCherry images were overlaid and ImageJ cell counter extension used to count the total number of GFP-positive cells in a section and GFP/mCherry-positive cells. Transduction efficiency of GFP-positive cells was calculated as: (total number of GFP cells co-expressing mCherry/total number of GFP cells) × 100. Mean transduction efficiency was determined from five to six sections per animal. The final mean was calculated across the three biological replicates and presented with the standard error of the mean.

### Dissection of M1 for RNA Extraction

Four weeks after surgery the lentiviral injected mice (P49–53) were anesthetized with pentobarbitol (150 mg/kg) and perfused intracardially with ice cold 0.9% (w/v) NaCl. One-millimetre coronal sections of the adult mouse brain were then made from Bregma 1.2–0 using a Ted Pella acrylic mold (Ted Pella Inc., Redding, CA, USA). Brain slices were transferred to L15 complete media containing phenol red free Leibovitz’s L15, 6% D-(+)-glucose (Sigma Aldrich, New Zealand) and 500 units penicillin/500 μg streptomyocin per mL (Invitrogen, New Zealand). A scalpel blade was used to dissect M1, including all layers and meninges, from coronal sections and an Olympus SZX12 with a Stereo Microscope Fluorescence Adapter with a 510–540 nm excitation lamp and 600 nm longpass emission filter (Nightsea, Lexington, MA, USA) used for the detection of mCherry-positive tissue. The dissected tissue was immediately flash frozen in liquid nitrogen.

### Preparation of RNA Samples from Mouse M1 Tissue for Transcriptomic Analysis

Frozen tissue was homogenized in 1 mL TRI^®^ reagent (Sigma-Aldrich, New Zealand) using an RNase free plastic pestle. Tissue debris was removed by centrifugation before adding chloroform (0.2 mL) to the supernatant and mixing vigorously for 15 s. After a 2–3 min incubation at room temperature, samples were centrifuged (12,000 × *g*) for 15 min at 4°C and the top aqueous phase removed to a fresh 1.5 mL eppendorf. This sample was then used as input to the Purelink^®^ RNA extraction kit (Life Technologies, New Zealand) and RNA eluted in 30 μL of RNase/DNase free H_2_O.

Before library generation for RNA-seq, the RNA was further purified using the RNA clean and concentrator™-5 (Zymo Research, Irvine, CA, USA). RNA quality was assessed using an Agilent Bioanalyzer™ and all samples had RIN numbers of 8.1 or higher. Libraries were prepared and sequenced from RNA samples by the Otago Genomics and Bioinformatics Facility. A TruSeq Stranded Total RNA Library Prep Kit (Illumina) was used and paired-end RNA-sequencing was performed on a HiSeq 2500 Illumina platform.

### Bioinformatics

Read quality was assessed using FastQC (Babraham Institute) and reads were trimmed of universal Illumina adapter sequence using fastq-mcf (Aronesty, 2011). Reads were mapped to the mouse genome build mm10 using tophat2 (Kim et al., 2013) and SAMTools (Li et al., 2009) was used to sort and index the resulting alignment bam files (mapped reads). Ensembl gene annotations were downloaded from the University of California Santa Cruz (UCSC) genome browser (January 2016) in BED format and read counts were assigned using BEDTools multiCov (Quinlan and Hall, 2010).

Differential expression analysis was performed using DESeq2 (version 1.10.1; Love et al., 2014) in R studio (R version 3.2.5; RStudio Team, 2015). First, genes with low coverage were filtered out using the cpm function of the edgeR package (Robinson et al., 2010). Only genes with >8 cpm in at least four samples were included in downstream differential expression analysis. Differentially expressed genes were selected with a false discovery rate (FDR) cutoff ≤0.05 and log fold change (LFC) cutoff ≥± 0.2.

Count data was transformed using the variance stabilizing transformation function in DESeq2 before clustering analysis. Unsupervised hierarchical clustering analysis was performed using R (version 3.2.5); first the top 8% most variable genes were identified across all eight samples, irrespective of treatment and a Euclidean distance matrix was produced using R package—Another Multidimensional Analysis Package (Lucas, 2009). The matrix was used as the input for unsupervised hierarchical clustering, which clusters samples according to the level of similarity identified by the distance matrix.

For heatmap generation the normalized counts were transformed using the DESeq2 variance stabilizing transformation and the z-score calculated for each sample. The heatmap was then generated using the heatmap.2 function in R’s (version 3.2.3) gplots package. The MA plot was created in R Studio (R version 3.2.3) by plotting the log10 normalized base mean output from DESeq2 against the LFC.

To represent the differentially repressed and activated genes in development (Lodato et al., 2014) and mature brain (our data), we used Network Analyst (Xia et al., 2015) to generate a chord diagram.

### Gene Ontology Analysis

Gene ontology and functional clustering analysis of all differentially expressed genes in mature M1 was performed using the online Database for Annotation, Visualization and Integrated Discovery (DAVID v6.7) tool: functional annotation (Huang et al., 2007). Kyoto Encyclopedia of Genes and Genomes (KEGG) pathway analysis, gene ontology analysis of mature-specific and development-specific (Lodato et al., 2014) *Fezf2*-regulated genes and mammalian phenotype analysis was performed using WebGestalt (Wang et al., 2013). The details can be found in the Supplementary Methods.

### Reverse Transcription and Quantitative PCR

RNA was treated with DNase I prior to cDNA synthesis. Five-hundred nanograms of RNA was added to 1 × DNase I buffer and 2 U of DNase I (Quantabio, Beverly, MA, USA) in a 10 μL reaction. After gentle mixing the reaction was incubated at 37°C for 30 min. One microliter of 10× stop buffer was added and incubated at 65°C for 10 min to inhibit DNase I activity. For cDNA synthesis, 60 μM of random hexamer primers were added to the DNase I treated RNA and incubated at 65°C for 10 min. A reaction mix was prepared with 200 nM dNTPs, 1× reaction buffer and 20 U RNase inhibitor (Roche, New Zealand) and added to each sample. Reverse transcriptase (RT; Roche, New Zealand) was then added (22 U) to each sample with the exception of the –RT negative control. Samples were then incubated at 55°C for 30 min then 85°C for 5 min before cooling samples to 4°C. The resulting cDNA was stored at −20°C.

Quantitative PCR was performed using a Roche LightCycler^®^ 480 SYBR green system. For relative quantification, target genes were amplified from cDNA samples alongside three stable reference genes pre-validated using refFinder (Xie et al., 2012). All primers were designed to span an exon-exon boundary using Primer-BLAST (Ye et al., 2012; Supplementary Table S1). Each reaction contained 3 μL of cDNA (diluted 1:9 in RNase/DNase free H_2_O), 500 nM of each forward and reverse primer and 1 × LightCycler 480 SYBR Green I master mix (Roche, New Zealand) made to 10 μL with RNase/DNase free H_2_O. Reactions were loaded in triplicate in a LightCycler^®^ 480 Multiwell −96 or −384 plate (Roche, New Zealand). A no template H_2_O and –RT negative control reaction was included on each plate for each primer set. The amplification protocol included enzyme activation 95°C for 5 min followed by 50 cycles of 95°C (5 s), 60°C (5 s), 72°C (10 s). Primer efficiencies (Supplementary Table S1) were determined from a standard curve of known cDNA input using the LightCycler^®^ 480 software.

Before relative quantification, standard deviation (SD) was calculated for all triplicates and when a single outlier was >2 SD from the mean it was removed. Mean Cp values were then used for relative quantification using the Pfaffl’s (2001) method, with the ratio of the target gene expressed relative to the geomean of all reference genes.

### Statistics

All statistical tests were performed using Prism 6 (Graphpad Software Inc., La Jolla, CA, USA). Unless stated otherwise, a two-tailed *t*-test was used for statistical analysis of mouse qPCR data. In the case of uneven variance a Mann-Whitney U test was applied. For all statistical analysis in the body of text the average is shown with standard error of the mean.

## Results

### Lentiviral-Mediated Delivery of shRNA Constructs to the Mouse Primary Motor Cortex (M1)

**Figure 1:**
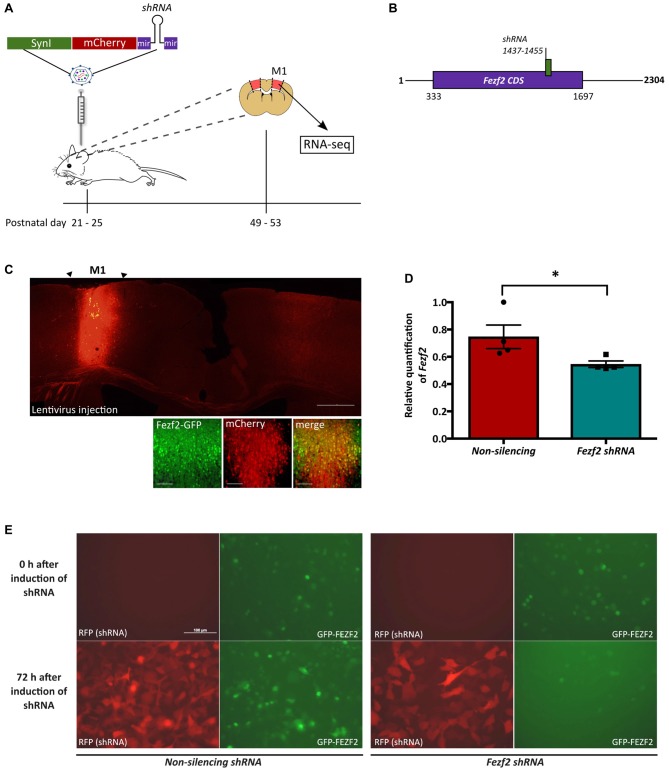
Lentiviral-mediated knockdown of *Forebrain embryonic zinc finger 2* (*Fezf2*) in the mature motor cortex. **(A)** Male mice aged P21–25 were injected into the primary motor cortex (M1) with lentivirus (LV) expressing a mCherry reporter gene and either a *Fezf2 shRNA* or *non-silencing shRNA*. Four weeks post-injection M1 tissue was isolated according to mCherry expression and processed for RNA-seq analysis. **(B)** Schematic indicating the binding site for the *Fezf2 shRNA* in the *Fezf2 mRNA*. **(C)** Injection of *LV.Syn.mCherry.non-silencingshRNA* in M1 of *Fezf2-Gfp* mice validated the successful transduction of *Fezf2*-expressing neurons. Brain section is from Bregma +0.4. Scale bar is 500 μm or 50 μm (higher magnification images). **(D)** Quantitative PCR analysis was used to test the *shRNA-*mediated knockdown of *Fezf2* expression in the mature M1 of wildtype male mice (*n* = 4, 4; *p < 0.05, unpaired one-tailed *t*-test). **(E)** Stable HT1080 cells transduced with a green fluorescence protein (GFP)-mFEZF2 fusion construct were induced to express either the *Fezf2 shRNA* or *non-silencing shRNA* (control). Images indicate *shRNA* expression according the RFP reporter and GFP-mFEZF2 expression (GFP) at 0 h and 72 h after the induction of shRNA expression. Scale bar is 100 μm.

We recently identified *Fezf2* expression in diverse pyramidal neuronal subtypes in layer 5 of the mature M1, including PT-PNs and IT-PNs (Tantirigama et al., 2016). We therefore wanted to reduce *Fezf2* expression globally within the mature M1, in order to target all *Fezf2*-expressing neurons (Figure 1A). As FEZF2 plays an essential role in the developing forebrain (Molyneaux et al., 2005; Hirata et al., 2006; Srinivasan et al., 2012), it was necessary to conditionally disrupt expression in the mature brain. To spatially and temporally restrict *Fezf2* knockdown to mature neurons of M1, we used lentiviral mediated-delivery of a *mCherry-Fezf2 shRNA* or a control *mCherry-non-silencing shRNA* construct under the regulation of the *Synapsin I* promoter (*LV.SynI.mCherry.shRNA*; Figures 1A,B). The spread of the virus and targeting of *Fezf2*-expressing neurons *in vivo* was first tested with the injection of *LV.Syn.mCherry.non-silencingshRNA* in three transgenic *Fezf2-Gfp* males. Brain sections were analyzed 4-weeks post-injection and the expression of mCherry was observed 1 mm (± 0.13, *n* = 3) rostral-caudal, inclusive of the two injection sites (Figure 1C). Of the *Fezf2*-GFP positive cells, 63% (± 1.65, *n* = 3) were transduced (mCherry-positive).

When injecting the *Fezf2 shRNA* into the M1 of *Fezf2-Gfp* mice, no changes in *Fezf2* expression were observed (data not shown). We previously demonstrated an increase in *Fezf2 CDS* expression in the *Fezf2-Gfp* mouse model (Tantirigama et al., 2016), which could have affected the shRNA efficiency. Therefore we chose to complete *Fezf2* knockdown experiments in wildtype mice. Male Swiss Webster mice (P21–25) were injected in M1 with either *LV.Syn.mCherry*.*Fezf2shRNA* LV or *LV.Syn.mCherry*.*non-silencingshRNA* (*n* = 4 of each; Figure 1A). At 4 weeks post-injection, *Fezf2*-expression was significantly decreased by 27% from 0.75 ± 0.09 (*n* = 4) in the *non-silencing shRNA-*treated control M1 samples to 0.55 ± 0.02 (*n* = 4) in the *Fezf2 shRNA*-treated samples (*p* = 0.03, one-tailed *t-test*; Figure 1D).

The lack of a good FEZF2 antibody prevented direct analysis of decreased protein expression in the M1 tissue. We therefore transduced a GFP-mFEZF2 fusion protein into HT1080 cells to monitor the efficacy of the *Fezf2 shRNA* on reducing protein levels. Qualitative analysis demonstrates a clear reduction in GFP expression at 72 h after the induction of *Fezf2 shRNA* expression in cells, whilst no reduction was observed in cells expressing the control *non-silencing shRNA* (Figure 1E). Additionally, the induction of *Fezf2 shRNA* expression before transduction of GFP-mFEZF2 maintains reduced GFP levels between 24 h and 72 h of expression (Supplementary Figure S1).

To control for potential off-target effects, the shRNA was checked for specificity. The *Fezf2 shRNA* showed 100% homology match to mouse *Fezf2* using basic local alignment search tool nucleotide (BLASTN, Altschul et al., 1997). There was additional alignment to mouse *Zfp523* with a much lower query cover of 73% (Supplementary Table S2). It is considered that any sequence ≥16 nt match (84%) to an off-target sequence would need to be discarded (Moore et al., 2010). As this non-specific alignment has a lower percentage of query cover the shRNA is unlikely to be able to bind to and produce mRNA cleavage.

### RNA-Sequencing of *Fezf2 shRNA*-Treated M1 Tissue Reveal Significant Changes in the Transcriptome Profiles

**Figure 2:**
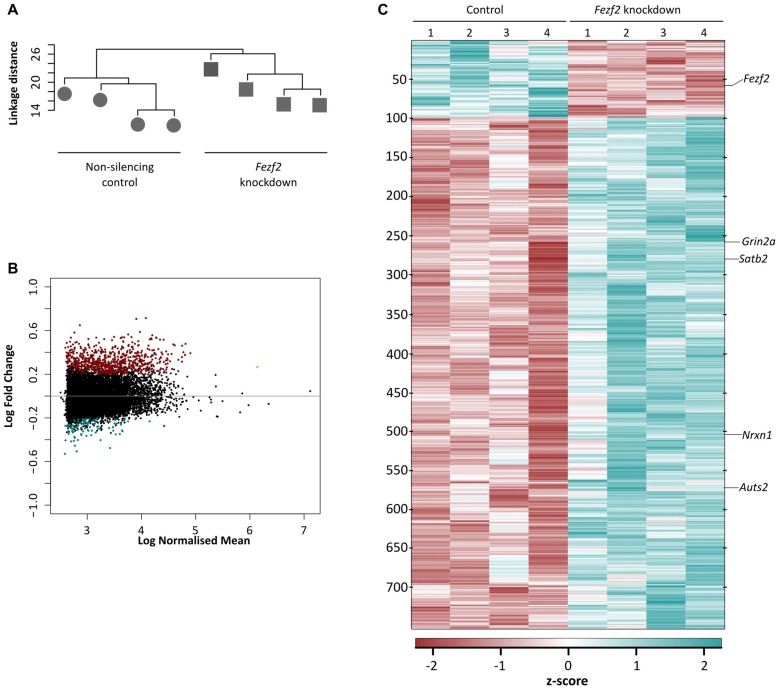
Conditional knockdown of *Fezf2* leads to significant changes in the transcriptome profile of M1. **(A)** Unsupervised hierarchical clustering of the top 8% most variable genes separated the control (*non-silencing shRNA*; circle) and *Fezf2-shRNA* treated (square) M1 tissue samples. **(B)** Heatmap shows the z-score from the transformed counts of each individual sample replicate, for all differentially expressed genes (false discovery rate (FDR) ≤ 0.05; log fold change (LFC) ≥ ± 0.2) identified in *Fezf2*-reduced M1. **(C)** MA plot shows (log10) normalized mean of all genes expressed across the M1 samples, plotted against the LFC. Upregulated genes (FDR ≤ 0.05; LFC ≥ 0.2) are in red and downregulated genes (FDR ≤ 0.05; LFC ≥ −0.2) are in blue.

As *Fezf2* is a transcription factor, if it continues to have a role in mature tissue, we hypothesized that even a small change to *Fezf2* expression would result in changes to the gene expression profiles of *Fezf2*-reduced M1 tissue. In order to investigate this, we used RNA-seq to generate transcriptome profiles of the control and *Fezf2 shRNA*-treated M1 tissue (*n* = 4 of each). For each library ~55 million total reads were generated. Concordant pair mapping rates to the mouse genome (mm10) were >89% (Table 1), with <8% showing >20 multiple alignments. To ascertain whether *Fezf2* knockdown could lead to reproducible changes in the M1 transcriptome, we then performed unsupervised hierarchical clustering analysis on the most variably expressed genes across all *Fezf2 shRNA*-treated and *non-silencing shRNA* treated M1 samples, irrespective of their treatment. Accordingly, two main sample clusters were identified, separating samples into knockdown and control groups (Figure 2A).

**Table 1:** Mapping rate of reads generated from RNA-sequencing of *Fezf2 shRNA* (*Fezf2* KD) or *non-silencing shRNA* (control) treated tissue.

	Number of paired raw reads	Number of paired reads after QC	% paired reads after QC	Number of aligned paired reads	% concordant pair alignment
Control 1	27709366	27709255	99.99	25261582	90.4
Control 2	26807763	26807639	99.99	24370739	90.5
Control 3	27120622	27120483	99.99	24368801	89.1
Control 4	26665873	26665772	99.99	24233182	90
*Fezf2* KD 1	28175979	28175862	99.99	25454116	89.5
*Fezf2* KD 2	27872058	27871935	99.99	25199887	89.9
*Fezf2* KD 3	27738010	27737896	99.99	24973199	89.6
*Fezf2* KD 4	27358847	27358749	99.99	24522056	88.9

**Figure 3:**
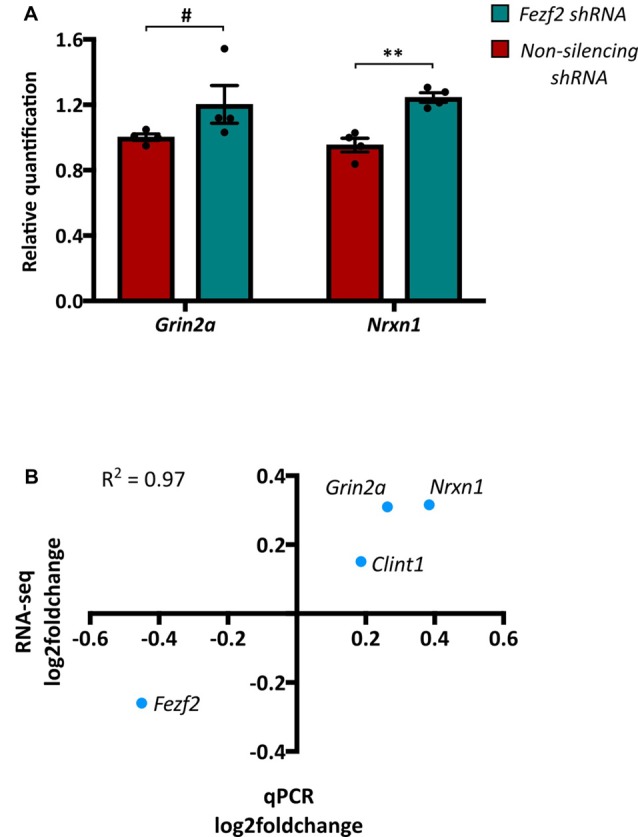
Quantitative PCR validation of RNA-seq data. **(A)** Two genes, *Grin2a* and *Nrxn1*, that were significantly changed between the *Fezf2 shRNA* and *non-silencing shRNA* treated M1 tissue, were validated using qPCR (*n* = 4, 4; **p < 0.01, unpaired two-tailed *t*-test; ^#^*p* < 0.1, Mann-Whitney U test). **(B)** Pearson’s correlation analysis was used to determine the correlation between the LFC values of genes validated by qPCR and those identified by RNA-seq (*R*^2^ = 0.97; *p* < 0.05).

Having identified a clear separation in the transcriptome profiles of control (*non-silencing shRNA*-treated) and *Fezf2 shRNA*-treated M1 tissues, we next wanted to determine which gene changes underpinned the sample clustering. We performed DESeq2 differential expression analyses on the M1 transcriptomes and revealed 756 genes to have significantly changed gene expression with the knockdown of *Fezf2* (FDR ≤ 0.05, LFC ≥ ± 0.2; Figure 2B heatmap; Supplementary Table S3), suggesting a regulatory role for *Fezf2* in the mature M1. The MA plot shown in Figure 2C highlights the genes significantly up- and down-regulated with *Fezf2* knockdown (FDR ≤ 0.05, LFC ≥ ± 0.2). For technical validation of RNA-seq, qPCR was used to analyze two randomly selected significantly changed genes, *Nrxn1* (*p* = 0.0013) and *Grin2a* (*p* = 0.0571; Figure 3A), and one gene that did not show significant differential expression, *Clint1* (*p* = 0.38). The LFC values obtained from the qPCR data of these three genes and *Fezf2* together displayed a significant positive correlation with the LFCs identified from RNA-seq analysis (*R*^2^ = 0.97, *p* < 0.05; Figure 3B), demonstrating technical validation of the RNA-seq platform.

To further corroborate that the observed changes in gene expression were caused by the reduction of *Fezf2*, we compared our *Fezf2*-regulated gene list to previously identified direct targets of FEZF2 (Chromatin immunoprecipitation sequencing data; Lodato et al., 2014). Comparisons revealed 65% of the significantly changed genes in mature M1 (FDR ≤ 0.05, LFC ≥ ± 0.2) are direct targets of FEZF2 (Supplementary Table S4), this percentage was similar amongst the *Fezf2*-regulated genes identified in development (67%; Lodato et al., 2014). Amongst the differentially expressed genes there were known developmental targets of FEZF2, including *Satb2* (LFC = 0.26, FDR = 0.02; Figure 2B) and *Auts2* (LFC = 0.45, FDR = 0.001; (Srinivasan et al., 2012); Figure 2B). With a number of the deregulated genes in the *Fezf2-shRNA* treated M1 not known to be direct targets, and several such as *Satb2* and *Auts2*, being transcription factors themselves, it is likely that many of the genes in this dataset could be indirectly affected by the knockdown of *Fezf2* expression.

### Comparison of *Fezf2*-Regulated Genes Identified in Developing and Mature Cortical Tissues Reveal a Change in Function

**Figure 4:**
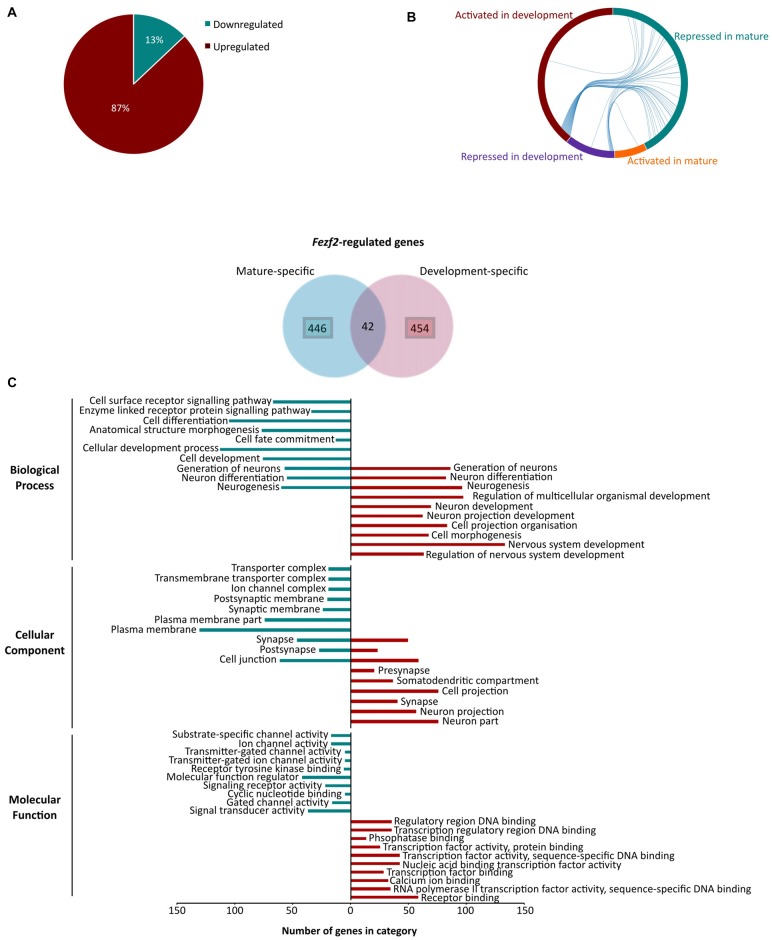
Changes in *Fezf2*-regulated genes between the developing and mature cortex indicate a switch in function for *Fezf2*. **(A)** Analysis of the differentially expressed gene list revealed that most were significantly upregulated (green) after *Fezf2* knockdown. **(B)** Chord diagram shows *Fezf2*-regulated genes common to both developing (Lodato et al., 2014) and mature tissues. Each line represents a gene and connects the groups in which the gene is present (mature repressed, blue; mature activated, orange; development repressed, purple and development activated, red). **(C)** Venn diagram shows *Fezf2*-regulated genes in mature and developing tissue that are known direct targets of FEZF2 (Lodato et al., 2014). To look for differences in the temporal specific *Fezf2*-regulated genes, gene ontology analysis using WebGestalt was performed on the two groups individually. The number of genes associated with the top 10 significantly enriched GO terms are plotted for mature- (blue) and development-specific (red) genes.

Intriguingly, of the significantly changed genes only 98 had decreased expression (13% of all genes), whilst 658 displayed an increase in expression (87%) after *Fezf2* knockdown (Figure 4A). These results indicate that *Fezf2* acts predominantly as a transcriptional repressor in the mature M1, whether directly or indirectly. Conversely, microarray analysis of *Fezf2* overexpression in the developing cortex indicated a prominent activator role for *Fezf2* (79% induced by Fezf2; Lodato et al., 2014). We were therefore interested in comparing our dataset of *Fezf2*-regulated genes (488 genes) to those identified previously in developing cortical neurons (496 genes; Lodato et al., 2014), focusing specifically on the known direct targets of FEZF2. Our comparison revealed an overlap of only 42 genes between these two datasets (Figure 4B; Supplementary Table S5). All genes activated by *Fezf2* in development were repressed in mature tissue (Figure 4B) and of the genes repressed by *Fezf2* in development only one became a target of transcriptional activation in mature tissue (*Gas5*; Figure 4B). The small overlap in *Fezf2*-regulated genes between developing and mature settings indicates a distinct change in role for FEZF2, which is further supported by the predominant switch in role from activator to repressor.

As there was only a small overlap in the *Fezf2*-regulated genes, we were interested to investigate whether the uniquely expressed genes indicate a change in function, specific to developing or mature neurons. To do this, we performed GO analysis on the *Fezf2*-regulated genes changed only in development or mature tissue. The top 10 significant biological processes enriched in the development-specific genes indicated a function in nervous system development, including neuron projection development (Figure 4C; Supplementary Table S6). This was corroborated by the cellular component (CC) terms, which were significantly enriched for localization to neuron or cell projection part, suggesting an importance for axonal targeting (Figure 4C; Supplementary Table S6). Analysis of the top enriched molecular function (MF) terms revealed an enriched role for *Fezf2*-regulated genes in transcription factor activity (Figure 4C; Supplementary Table S6), including factors such as *Ctip2* (*Bcl11b*) that are important for the specification of layer-restricted PN types. This highlights FEZF2’s known key role in the regulatory genetic networks, which direct the correct development of the layered cortex (Srinivasan et al., 2012).

Comparative analysis of the mature M1-specific genes revealed some overlap in the biological process terms of the development-specific genes (e.g., neuron differentiation, neurogenesis), however unique terms identified in this dataset included receptor signaling pathways (Figure 4C; Supplementary Table S6). Accordingly, analysis of the CC terms amongst the mature-specific genes revealed enrichment of localization to the synaptic and postsynaptic membranes as well as ion channel complexes (Figure 4C; Supplementary Table S6). Moreover, analysis of MF terms indicated a unique enrichment of roles in the regulation of ion channel activity amongst the mature-specific genes (Figure 4C; Supplementary Table S6). This term enrichment analysis indicates an important functional role for the *Fezf2*-regulated mature-specific genes in the transmission of signals and movement of ions, important functions for mature neuron activity.

Together these gene ontology analyses indicate a distinct role for unique *Fezf2*-regulated genes in the developing and mature cortex, relevant to the environmental setting. This could suggest that the divergence in *Fezf2’s* role is influenced by additional genetic or epigenetic factors that change between development and maturity.

### KEGG Pathway Analysis of *Fezf2*-Regulated Genes

**Figure 5:**
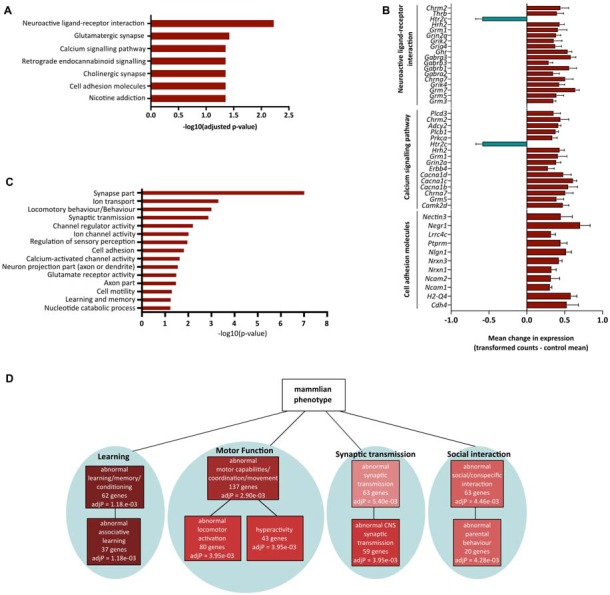
Functional annotation of significantly altered genes after *Fezf2* knockdown in mouse M1. **(A)** KEGG pathway analysis of *Fezf2*-regulated genes. Seven pathways were significantly enriched (−log10 adjusted *p*-value ≥ 1.3). **(B)** Mean change in gene expression (±SEM) for pathway-associated (neuroactive ligand receptor interaction, calcium signaling pathway and cell adhesion molecules) genes differentially expressed in *Fezf2*-reduced M1. Relative expression is calculated as the transformed counts for each *Fezf2*-reduced M1 sample—the mean transformed counts of the control samples. **(C)** Analysis of all the *Fezf2*-regulated genes using Database for Annotation, Visualization and Integrated Discovery (DAVID) revealed 15 clusters with significant enrichment (−log10 *p*-value ≥ 1.3). Red dashed line indicates cut-off for significance. **(D)** Overrepresentation analysis of *Fezf2*-regulated genes using the mammalian phenotype ontology database. Of the top 10 significant phenotypes enriched four main groups were identified; learning, motor function, synaptic transmission and social behavior. Key indicates significance (adjusted *p*-value ≤ 0.05).

Having demonstrated significant molecular effects of reducing *Fezf2* in the mature M1, and a unique role for the direct FEZF2 targets in mature tissue, we were interested in investigating further the functional role for all *Fezf2*-regulated genes. We first used the KEGG pathway database to investigate whether the *Fezf2*-regulated genes were statistically enriched for specific biochemical pathways. Amongst the 756 differentially expressed genes there was significant enrichment of seven KEGG pathways (adjusted *p*-value ≤ 0.05; Figure 5A; Table 2).

**Table 2:** Results from Kyoto Encyclopedia Genes and Genomes (KEGG) pathway analysis of *Fezf2*-regulated genes.

KEGG ID	Name	FDR	Number of genes	Gene symbol
mmu04080	Neuroactive ligand-receptor interaction	5.62E-03	18	*Grm3, Grm5, Grm7, Grik4, Chrna7, Gabra2, Gabrb1, Gabrb3, Gabrg3, Ghr, Gria4, Grik2, Grin2a, Grm1, Hrh2, Htr2c, Thrb, Chrm2*
mmu04724	Glutamatergic synapse	3.58E-02	16	*Grm3, Grm5, Grm7, Grik4, Cacna1c, Cacna1d, Gria4, Grik2, Grin2a, Grm1 Prkca, Plcb1, Adcy2, Shank2, Dlgap1, Homer2*
mmu04020	Calcium signaling pathway	4.18E-02	16	*Camk2d, Grm5, Chrna7, Cacna1b, Cacna1c, Cacna1d, Erbb4, Grin2a, Grm1, Hrh2, Htr2c, Prkca, Plcb1, Adcy2, Chrm2, Plcd3*
mmu04725	Cholinergic synapse	4.18E-02	14	*Camk2d, Kcnq3, Ache, Chrna7, Cacna1b, Cacna1c, Cacna1d, Fyn, Kcnq1, Prkca, Plcb1, Adcy2, Kcnq5, Chrm2*
mmu04723	Retrograde endocannabinoid signaling	4.18E-02	14	*Grm5, Cacna1b, Cacna1c, Cacna1d, Gabra2, Gabrb1, Gabrb3, Gabrg3, Gria4, Grm1, Prkca, Plcb1, Adcy2, Abhd6*
mmu04514	Cell adhesion molecules (CAMs)	4.18E-02	11	*Cdh4, H2-Q4, Ncam1, Ncam2, Nrxn1, Nrxn3, Nlgn1, Ptprm, Lrrc4c, Negr1, Nectin3*
mmu05033	Nicotine addiction	4.18E-02	8	*Chrna7, Cacna1b, Gabra2, Gabrb1, Gabrb3, Gabrg3, Gria4, Grin2a*

The top significantly overrepresented pathway was neuroactive ligand-receptor interaction (Figure 5A; Table 2). Similarly, there was significant enrichment of the glutamatergic synapse, cholinergic synapse, retrograde endocannabinoid signaling and nicotine addiction pathways (Figure 5A). Together, these pathways included genes important for neurotransmitter signaling, movement of ions at the membrane and synaptic scaffolding (Table 2). The enrichment of this group of pathways provides evidence of a functional role for *Fezf2* in regulating neuronal signaling at the synapse.

In addition to the general role for *Fezf2*-regulated genes in neuronal transmission, two other pathways were significantly overrepresented in this gene set. Sixteen *Fezf2*-regulated genes were identified in the calcium-signaling pathway (Figure 5A; Table 2), with the dysregulated genes predominantly involved in the calcium transport at the membrane (5/16 genes; *Cacna1b, c, d*, *Grin2a* and *Chrna7*) or triggering intracellular calcium release (9/16 genes; *Grm1, Erbb4, Grm5, Htr2c, Prkca, Plcb1, Adcy2, Chrm2* and *Plcd3*). The nature of the enriched genes in the calcium signaling pathway therefore suggests an importance for *Fezf2* in the regulation of calcium flux in neurons, which is important to the plasticity of neurons (Adasme et al., 2011).

Finally, 11 genes were identified in the cell adhesion molecules pathway. Most cell adhesion molecules changed here after *Fezf2* knockdown are associated with synaptic interactions (7/11; *Ncam1, Ncam2, Nrxn1, Nrxn3, Nlgn1, Negr1* and *Nectin3*), providing further support to the role for *Fezf2* in the regulation of neuronal transmission. The remaining genes were implicated in either neuronal growth cone and axon association (*Ptprm* and *Lrrc4c*) or the immune system (*H2-Q4*; Table 2).

Analysis of the gene expression changes in these pathways revealed nearly all but one of the *Fezf2*-regulated genes as up-regulated (Figure 5B). These analyses together indicate a negative regulatory role for *Fezf2* in neuronal signaling and plasticity in the mature M1.

### Gene Ontology Analysis of *Fezf2*-Regulated Genes

To further investigate enrichment of functional roles for the *Fezf2*-regulated genes in mature M1, we performed functional annotation clustering based on gene ontology classes using DAVID (Huang et al., 2007). Fifteen significantly overrepresented functional clusters were found (Figure 5C; Supplementary Table S7), which together additionally suggests a functional role for *Fezf2* in mediating neuronal transmission and function. For example, clusters included an enrichment of genes involved in synaptic transmission, regulation of ion channel activity and encoding mRNAs that localize to the synapse or axon part (Figure 5C).

In addition to the generalized functional role for *Fezf2* in neuronal activity, a significant functional cluster showed an enrichment of genes important for regulating behavior (locomotory, sensory perception and learning/memory; Figure 5C). To examine whether these behavior-related genes have a direct association with specific known phenotypes we performed overrepresentation analysis using the mammalian phenotype ontology database (Smith et al., 2004). Amongst the top 10 significantly overrepresented phenotypes four main groups were identified, abnormal learning/memory, abnormal motor capabilities, abnormal social interaction and abnormal synaptic transmission (Figure 5D; Supplementary Table S8), similar to the findings from the DAVID analysis. However, the use of this database also identified significant overrepresentation of specific behavioral phenotypes associated with these main groups, including abnormal associative learning, hyperactivity and abnormal locomotor activation (Figure 5D; Supplementary Table S8). Based on these results maintaining *Fezf2* expression could therefore be functionally important to the normal regulation of behavior, particularly learning and motor behavior.

## Discussion

During development, *Fezf2* has a defined function regulating the expression of genes important to the correct specification of axonal projection and directing the excitatory fate of CSpPNs in layer 5 of the cortex (Chen B. et al., 2005; Chen J.-G. et al., 2005; Molyneaux et al., 2005; Lodato et al., 2014). Although there is significant understanding of the regulatory role FEZF2 plays in cortical development (Molyneaux et al., 2005; Srinivasan et al., 2012; Guo et al., 2013), the impact of changing *Fezf2* gene expression levels in the mature brain has been largely neglected. In this study, we investigated the hypothesis that *Fezf2* expression in the mature M1 continues to have an important functional role for the regulation of gene expression. Here, our results demonstrate that reducing *Fezf2* expression, specifically in M1, causes significant changes to the transcriptome, altering the expression of genes that collectively have the potential to disrupt normal neuronal function and behavior.

Notably, our dataset reveals a predominant repressor role for *Fezf2* in mature M1 (Figure 4A), which is in contrast to the previously observed activator role for *Fezf2* in development (Lodato et al., 2014). In accordance with our findings, *FEZF2* is a known tumor suppressor gene in mature tissue, with the silencing of *FEZF2* expression causative of nasopharyngeal carcinoma and implicated in brain tumors (Shu et al., 2013). This contrast in regulatory function could indicate an important adaptive change for *Fezf2* based on tissue state. Certainly, our analyses of the *Fezf2*-regulated genes identified in developing and mature tissues revealed only a small subset common to both datasets and two distinct sets of genes that together have unique functional roles specific to each environmental setting (Figure 4C). Such a distinctive transition in *Fezf2* function could be indicative of an influence from changes in the chromatin landscape that are commonly observed through developing and differentiated cell states. For example, chromatin becomes more compact in differentiated cells (Chen and Dent, 2014), which could refine the binding sites available to FEZF2. However, of the *Fezf2*-regulated genes common to developing and mature tissue, there was a shift of all genes once activated by *Fezf2* to be repressed (Figure 4B). FEZF2 itself could therefore be important for regulating changes to the chromatin state, to allow a switch in activation to repression of the same gene. Transcription factors, once bound to the DNA, can regulate active or repressive changes to chromatin remodeling, dependent on the factors recruited (Brettingham-Moore et al., 2015). For example, transcription factor RUNX1, essential to hematopoietic differentiation, recruits protein arginine methyltransferase 6 (PRMT6) in progenitor cells, which mediates repressive epigenetic modifications to the genes required for differentiation. Once the progenitor cells differentiate, the association of RUNX1 with PRMT6 is lost and a coactivator complex recruited in its place (Herglotz et al., 2013). *Fezf2* interacts with members of the corepressor groucho family (Zhang et al., 2014), which can recruit additional factors such as PRMT5 to mediate repressive modifications to the chromatin (Patel et al., 2012). Increased availability and/or increased interaction with such cofactors could facilitate the FEZF2 repressor function in mature tissues.

Changes in gene expression are commonly observed in neurological disease (Wang et al., 2007; Satoh et al., 2014) and may represent negative changes in neural cells. For example, disruption to the expression of genes that dictate neuronal identity could contribute to the progression of neuropsychiatric and neurodegenerative disease (Kadkhodaei et al., 2009; Liu et al., 2010; Song et al., 2011; Deneris and Hobert, 2014; Jaitner et al., 2016). Our previous characterization of the functional phenotypes in *Fezf2*-expressing neurons of M1 identified a wider action potential in *Fezf2*-expressing neurons indicating an enhancement of calcium flux in these neurons (Tantirigama et al., 2014, 2016). It was therefore noteworthy to find an enrichment of *Fezf2*-regulated genes involved in the regulation of calcium flux (Figures 5A,B), a function directly relevant to *Fezf2*-expressing neuronal phenotypes. Nearly all the *Fezf2*-regulated genes identified in the calcium-signaling pathway here were upregulated with *Fezf2* knockdown, suggesting that *Fezf2* is a negative regulator of calcium flux. This negative regulation could have huge importance in maintaining healthy mature neurons, as excessive calcium signaling is a common trait in several neurodegenerative diseases, including ALS (Marambaud et al., 2009). Additional enriched pathways and Gene Ontology analysis also indicated a general functional importance for *Fezf2*-regulated genes in neuronal signaling and plasticity (Figures 5A,C). The regulation of synaptic transmission and cell signaling is essential to the phenotype of neurons and would therefore indicate a role for *Fezf2* in sustaining neuronal phenotypes in the healthy mature M1. It will be interesting to investigate the effects of these gene changes on the functional phenotypes of *Fezf2*-expressing neurons in M1.

Gene Ontology and Phenotype Ontology analysis of *Fezf2*-regulated genes additionally indicated a functional role in regulating behavior (Figures 5C,D), with specific behavior phenotypes including abnormal associative learning, abnormal social interaction, abnormal locomotor activation and hyperactivity (Figure 5D). As our work is the first main study of *Fezf2’s* role in the mature brain, there is currently no research investigating the effects of *Fezf2* disruption on adult behavior, though interestingly hyperactivity is a developmental behavioral phenotype observed in *Fezf2−/−* mice (Hirata et al., 2004). Our findings of enriched associations in the mature M1 *Fezf2*-regulated genes to highly specific behavior phenotypes indicate that temporal disruption to *Fezf2* expression could negatively impact normal behavior.

## Conclusion

Here we used lentiviral-mediated conditional knockdown and RNA-seq technologies to provide evidence of a regulatory role for *Fezf2* in the mature mammalian brain. Our findings strengthen the idea that active expression of transcription factors is critical for the genetic networks in a healthy adult brain. Through the comparison of genes regulated by *Fezf2* in developing and mature tissues we demonstrate dynamic changes in the functional role of *Fezf2*. Moreover, the overrepresentation analyses of pathway and phenotype datasets, for *Fezf2*-regulated genes in mature M1, emphasize the potential functional implications of disrupting these gene networks. Together this highlights an importance for identifying and understanding the role of regulatory factors in matured neuronal subtypes.

## Accession Code

RNA-seq data, GSE102365.

## Author Contributions

AJC, RME and SMH: study concept and design. AJC and HEW performed the experiments and acquired data. AJC, HEW, RME and SMH: analysis and interpretation of the data. AJC: drafting of the manuscript. AJC, SMH, RME and HEW: major critical revisions of the manuscript. All authors had full access to the manuscript and data for approval.

## Conflict of Interest Statement

The authors declare that the research was conducted in the absence of any commercial or financial relationships that could be construed as a potential conflict of interest.

## Abbreviations

CSpPNs: corticospinal projection neurons
EPA: environmental protection agency
*Fezf2*: Forebrain embryonic zinc finger 2
GFP: green fluorescence protein
IT-PN: intratelencephalic projection neuron
KEGG: Kyoto encyclopedia of genes and genomes
LV: lentivirus
M1: primary motor cortex
PNs: projection neurons
PT-PN: pyramidal tract projection neuron.
