# Supplementary material for: RNA-Sequencing Analysis Reveals a Regulatory Role for Transcription Factor Fezf2 in the Mature Motor Cortex
Source: Front Mol Neurosci. 2017 Sep 7;10:283. doi: 10.3389/fnmol.2017.00283 (PMC5594072; doi:10.3389/fnmol.2017.00283)
Supplement: Supplementary file 9 [file Data_Sheet_1.docx]

**Supplementary Material**

**RNA-sequencing analysis reveals a regulatory role for transcription factor *Fezf2* in the mature motor cortex**

Alison J. Clare^1,3,4^, Hollie E. Wicky^1,3,4^, Ruth M. Empson^2,3^ and Stephanie M. Hughes^1,3,4^*.

Departments of ^1^Biochemistry and ^2^Physiology, ^3^Brain Health Research Centre, ^4^Genetics Otago, School of Biomedical Sciences, University of Otago, Dunedin, New Zealand 9054.

*** Correspondence**:

Stephanie M. Hughes

[stephanie.hughes@otago.ac.nz](mailto:stephanie.hughes@otago.ac.nz)

**Supplementary Figures**

**
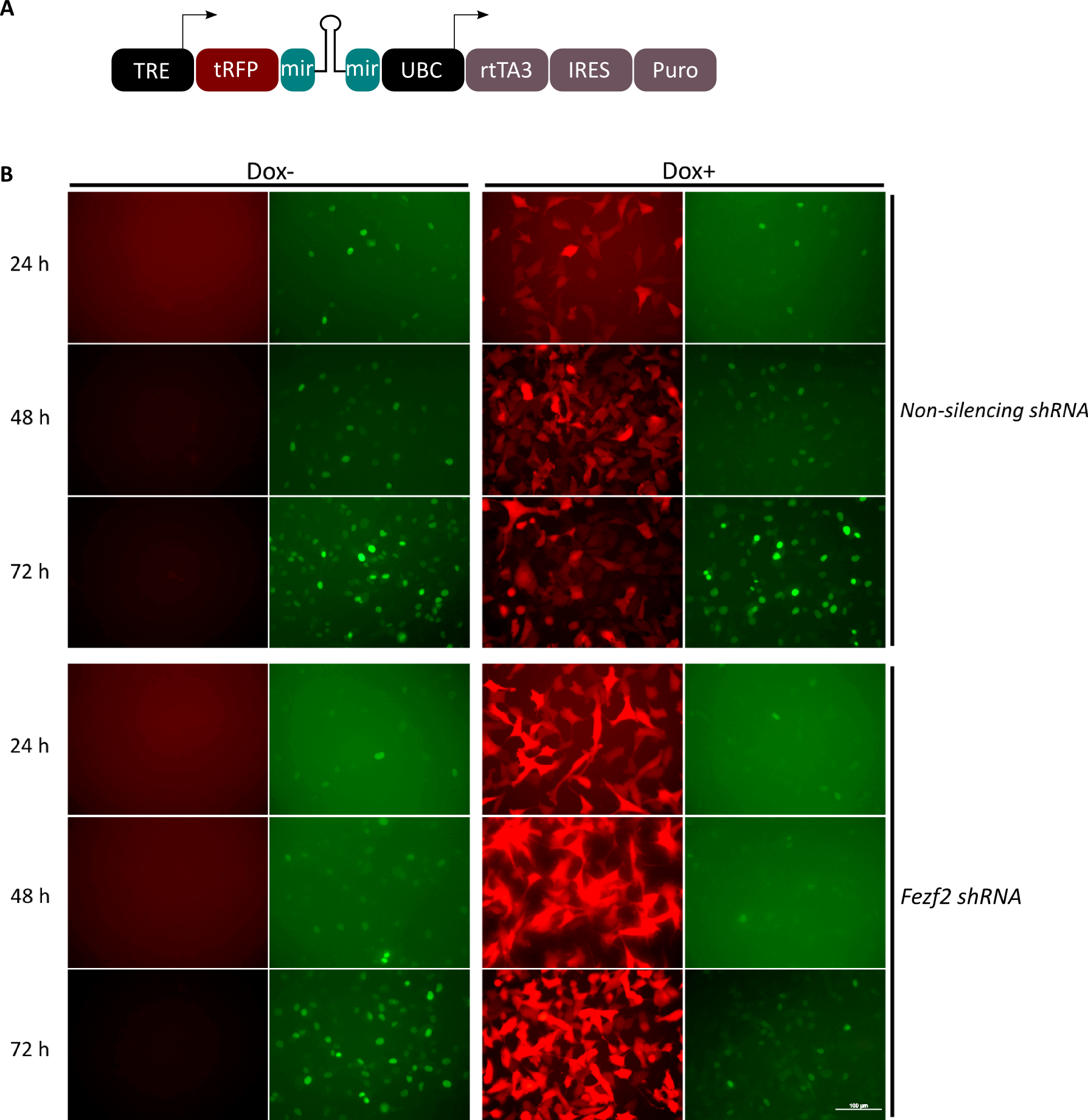
**

**Supplementary Figure 1 Mouse *Fezf2 shRNA* reduces GFP-FEZF2 protein expression in HT1080 cells. (A)** TRIPZ construct used to test the mouse *Fezf2 shRNA* effects on protein expression in HT1080 cells. Tetracycline response element (TRE) promotes expression of turbo RFP (tRFP) and shRNA (*Fezf2* or *non-silencing*) in the presence of tetracycline/doxycycline. A constitutive promoter, human ubiquitin C (UBC) regulates the expression of reverse tetracycline-transactivator 3 (rtTA3), required for tetracycline/doxycycline-dependent induction of TRE promoter, and puromycin resistance gene (Puro) used for selection of TRIPZ expressing cells in the generation of stable cell lines. The internal ribosomal entry site (IRES) allows the rtTA3 and Puro to be expressed as a single transcript. **(B)** Stable HT1080 cells were induced to express either the *non-silencing* or *Fezf2 shRNA* by addition of doxycycline (Dox+), before transduction of the *Gfp-Fezf2* construct. Uninduced cells (Dox-) were included as controls. RFP expression acts as a reporter of shRNA expression in the induced cells. GFP expression indicates the level of FEZF2 expression in cells. Scale bar is 100 µm.

**Supplementary Methods**

**Immunohistochemistry**

Sections were washed and thawed in phosphate buffer then blocked with PBS containing 3% (v/v) normal goat serum. We used a rabbit polyclonal anti-GFP (1:9999; Abcam, NZ; AB290) that first required a pre-incubation step on non-GFP expressing tissue for 1 h to reduce background from non-specific binding (Linterman *et al.* 2011). Tissue was then incubated overnight at 4 °C with anti-GFP and a rat monoclonal anti-dsRed primary antibody (1:999; ChromoTek [5F8]). Following three washes in PBS with Triton X-100 (0.2%) sections were incubated for 4 h at room temperature with Alexa Fluor 488 goat anti-rabbit secondary antibody (1:999; Invitrogen, NZ; A-11008) to enhance GFP signal and Alexa Fluor 555 conjugated goat anti-rat secondary antibody to enhance mCherry signal (1:999; Invitrogen, NZ; A-21434). After immunolabelling, sections were mounted on glass slides in anti-fade solution (0.1 g p-phenylenediamine made with 80% (v/v) glycerol in 0.1M phosphate buffer, pH 8.5). Fluorescent images were captured using the Olympus inverted microscope IX71 and merged using Adobe Photoshop. The brain regions that had fluorescence were identified using criteria from the Franklin and Paxinos Mouse Brain Atlas (Paxinos and Franklin, 2004).

**Validation of FEZF2 protein knockdown in cell culture**

HT1080 cells were maintained in Dulbecco’s modified Eagle’s medium (DMEM; Life Technologies, NZ) with 10% fetal bovine serum (Thermo Fisher Scientific, NZ), 100 units of penicillin/streptomycin, 2 mM L-glutamine, 1 mM sodium pyruvate and 1X non-essential amino acids (Life Technologies, NZ). To express and test the efficiency of the mouse *Fezf2 shRNA* construct in HT1080 cells a constitutively expressed promoter was required. We therefore cloned the mouse *Fezf2 shRNA* sequence into a pTRIPZ lentiviral plasmid backbone (RHS4743; Open Biosystems, GE Healthcare, Dharmacon, CO, USA), which expresses an *Rfp* reporter gene and *shRNA* under the regulation of a tetracycline/doxycycline inducible promoter (**Supplementary Figure 1A**). The pTRIPZ plasmid was the original source of the *non-silencing shRNA* used in this study. Stable HT1080 cell lines were then generated to express either the mouse *Fezf2* *shRNA* or *non-silencing shRNA*. Briefly, HT1080 cells were seeded at 5 x 10^4^ in a 24-well plate and between 1 – 20 µL of pTRIPZ *Fezf2 shRNA* or pTRIPZ *non-silencing shRNA* expressing concentrated lentivirus was added to achieve a multiplicity of infection (MOI) of 1 - 15. Cells expressing the construct were then selected for according to the expression of a puromycin resistance gene from the pTRIPZ construct (**Supplementary Figure 1A**), by the addition of 0.5 µg/mL puromycin (GIBCO, Invitrogen, NZ). For the induction of RFP and *shRNA* expression 2 µg/mL of doxycycline was added to the cell media. To analyse the efficiency of the *Fezf2 shRNA* in protein reduction, the stable cell lines were plated at 1.0 x 10^4^ in a 6-well plate before transduction with lentivirus expressing a mouse *Gfp-Fezf2* construct at an MOI of 8.6. In two separate experiments, the *Gfp-Fezf2* construct was transduced either 48 h prior to induction of *shRNA* expression or once the *shRNA* was already induced. The expression of GFP-FEZF2 and *shRNA* (RFP) was then monitored at 24 h, 48 h and 72 h, either post *Gfp-Fezf2* transduction or post *shRNA* induction. Images were captured at each time-point using a 40 x objective on an Olympus IX71 microscope.

**Validation of shRNA sequence specificity**

To confirm the specificity of the *Fezf2* sequence the National Centre for Biotechnology Information (NCBI) tool; basic local alignment search tool nucleotide (BLASTN, Altschul et al. 1997) was used to align the sequence specific portions of the shRNA to the mouse reference RNA sequence database.

**Gene Ontology analysis**

For both DAVID and WebGestalt analyses, all genes expressed across the M1 samples after filtering based on cpm were uploaded as the background gene list. When analysing the microarray dataset from Lodato *et al.* (2014), a list of expressed genes in all samples was acquired from Gene Expression Omnibus (GSE56451). For DAVID analysis of all Fezf2-regulated genes identified in mature M1, the categories databases included for term enrichment analysis were the three gene ontology categories (biological pathway, cellular component and molecular function). The standard cluster settings were applied; classification stringency, medium and EASE score, 1.0. The significance cut-off for each annotation cluster was based on an assigned enrichment score of 1.3 or higher. These scores are –log10 of the p-value (e.g. a score of ≥ 1.3 is equal to a p-value ≤ 0.05). Each individual term that makes up a cluster is additionally reported with a Benjamini-Hochberg adjusted p-value (**Supplementary table 5**). KEGG pathway analysis of *Fezf2*-regulated genes in mature M1 was performed on Webgestalt (Wang *et al.* 2013), with multiple testing correction by the Benjamini-Hochberg method. To identify overrepresentation of genes with known associated mammalian phenotypes, the mammalian phenotype ontology database (Smith *et al*. 2004) was used on Webgestalt (Wang *et al.* 2013) and the Benjamini-Hochberg multiple testing correction was applied (**Supplementary table 6**). For the analysis of term enrichment in *Fezf2*-regulated genes specific to developing (Lodato *et al.* 2014) and mature cortical tissue, gene ontology analysis was performed using WebGestalt (Wang *et al.* 2013), with Bentamini-Hochberg multiple testing correction. In advanced parameters an output of the top 10 significant gene ontology terms only was selected.
